# Proceedings of the 2020 American Bee Research Conference

**DOI:** 10.3390/insects11060362

**Published:** 2020-06-11

**Authors:** Bradley N. Metz, Judy Wu-Smart, Michael Simone-Finstrom

**Affiliations:** 1 American Association of Professional Apiculturists, Lincoln, NE 68583, USA; jwu-smart@UNL.EDU (J.W.-S.); Michael.SimoneFinstrom@usda.gov (M.S.-F.); 2 Department of Entomology & Plant Pathology, North Carolina State University, Raleigh, NC 27607, USA; 3 Department of Entomology, University of Nebraska, Lincoln, NE 68583, USA; 4 USDA-ARS, Honey Bee Breeding, Genetics and Physiology Research Laboratory, Baton Rouge, LA 70820, USA

**Keywords:** *Apis mellifera*, honey bee biology, apiculture, American Association of Professional Apiculturists

## Abstract

The 2020 American Bee Research Conference (ABRC) was held on 9–10 January 2020 in conjunction with the annual convention of the American Beekeeping Federation Conference and Trade Show in Schaumburg, IL. Over the two-day conference, a total of 65 oral and poster presentations were given, representing work done from over 30 different research groups from throughout the United States and Canada. These proceedings contain the submitted abstracts for presentations given at the 2020 American Bee Research Conference.

## 1. Introduction and Overview

The American Association of Professional Apiculturists (AAPA) held its annual American Bee Research Conference (ABRC) on 9–10 January 2020 at the Renaissance Schaumburg Convention Center Hotel in Schaumburg, IL. The conference was held in conjunction with the American Beekeeping Federation Conference and Trade Show (ABF) and the Apiculture Inspectors of America (AIA). With 65 oral and poster presentations given, research was presented from over 30 different research groups throughout the United States and Canada. The AAPA is pleased to present the submitted abstracts of many of the presentations given over the course of the two-day conference.

## 2. Abstracts of Presentations

### 2.1. The Role of Tree Lines as Pesticide Drift Barriers to Reduce Non-Target Exposure and Promote Healthy Pollinator Communities in Agricultural Landscapes

VakilSurabhi GuptaWu-SmartJudyDepartment of Entomology, University of Nebraska-Lincoln, Lincoln, NE, USA

Pollinators play a key role in providing vital ecosystem services that maintain biodiversity in fragile, diminishing, or degraded natural landscapes. Approximately 35% of the global food production relies on animal pollination, and 80% of wild plants rely on insect pollination most of which is provided by bees. In the last decade, there have been numerous reports of pollinator losses, such as wild bees (abundance has declined by 23%), commercially-managed honey bees (40% annual winter mortality), and monarch butterflies (abundance reduced by 15%). Major factors responsible for pollinator health decline include diminishing habitat and exposure to agrochemicals. To mitigate losses, recommendations promote pollinator habitats in agricultural, urban, and natural landscapes, however, improperly placed plantings may unintentionally expose pollinators to agrochemicals. Floral resources near cropping systems, for example, may become contaminated from off-target drift and act as a sink for pesticides unintentionally exposing foraging bees and other beneficial insects. Of concern are systemic neonicotinoid insecticides and *Bacillus thuringiensis* (Bt) toxin-based biocides used in corn production that may drift off crop fields and into pollinator habitat. This project examines the use of existing tree lines as potential drift barriers to mitigate neonicotinoid and Bt contamination of pollinator habitats established near crop fields. Neonicotinoid residues were collected using sticky traps near tree lines and from flowers within pollinator habitats with and without drift barriers to determine the field level exposure to bees. Similarly, leaves were collected from the milkweed plants growing at corn field margins to evaluate the exposure risk to Bt pollen and cry protein concentration. The field realized values will inform relevant dosing concentrations for supplemental laboratory toxicity bioassays. The abundance and diversity of bees were assessed to evaluate which insects are most at risk and the potential effects on pollinator communities. Results of this study will improve current recommendations for landscape management with special consideration to protecting pollinator resources in high agricultural production areas.

### 2.2. A Longitudinal Study of the Principle Factors Leading to Colony Losses in Migratory Beekeeping

O’Shea-WhellerThomas^1^Simone-FinstromMichael^2^DankaBob^2^RinkevichFrank^2^HealyKristen^1^PennHannah^1^SwaleDaniel^1^LangSarah^1^FellowsC.J.^1^1Department of Entomology, Louisiana State University, 404 Life Sciences Building, Baton Rouge, LA 70803, USA2USDA Agricultural Research Service, Honey Bee Breeding, Genetics, and Physiology Laboratory, Baton Rouge, LA 70820, USA

Commercial beekeeping in the United States accounts for >75% of all colonies in circulation, with migratory pollination comprising a large proportion of the industry. However, the system experiences high overwinter losses on a consistent and yearly basis, with preliminary data for this year placing losses at 39.9%. Consequently, without substantial improvements in colony health, large-scale migratory pollination is likely to become both biologically unsustainable, and commercially in-viable in its current form. Our project aims to identify and model the key predictors of colony loss, and their relative importance, both at a large spatiotemporal scale, and under real-world conditions. We demonstrate the relative weightings of parasite, disease, forage and pesticide stressors upon colony health. Crucially, our data show that *Varroa* accounts for ~70% of observed mortality across regions. We also demonstrate the success of an integrated management strategy, namely the use of mite-resistant ‘Pol-Line’ bees (Figure 1). This stock was found to experience a marked and significant reduction in mortality, and consistently lower mite levels throughout the year, presenting a viable alternative to current commercial stocks. In sum, our data suggest that *Varroa* should be the prime concern if colony losses are to be best addressed, and that integrated control methods, principally in the form of mite-resistant stocks, are the most promising long-term solution to the current *V. destructor* pandemic.

### 2.3. Using Honey Bee Foraging Choice to Understand Colony-Level Dietary Deficiencies

Corby-HarrisVanessaUSDA-ARS, Tucson, AZ, USA

Honey bees acquire the bulk of their dietary lipid requirements from pollen. Commercial honey bee colonies do not always have access to high-quality pollen, however, and so beekeepers must supplement hives with a commercially available diet. Unfortunately, these diets do not provide the complete profile of lipids to the colony, causing colony populations to decline precipitously after approximately six weeks. An important question in honey bee diet research is what makes these diets deficient and whether their nutritional value can be improved. Previous work suggests that honey bees (*Apis mellifera*) will preferentially forage for diets that rescue previous dietary deficiencies. Here, we seek to leverage information about foraging preference to test whether and how commercially available supplemental feeds are nutritionally deficient. We first measured the fatty acid content of several commercially available dietary supplements, identifying diets that were either high or low in an essential fatty acid. We then fed hives diets that had high or low levels of these fatty acids over a six-week period and asked whether they chose diets that rescued the prior deficiency. We discuss our results in the context of honey bee nutrition and in the context of developing more complete diets for honey bees.

### 2.4. A Two-Tier Method for Counting Omnidirectional Bee Motions in Video

KulyukinVladimirMukherjeeSarbajitDepartment of Computer Science, Utah State University, Logan, UT, USA

Omnidirectional bee traffic is the number of bees moving in arbitrary directions in close proximity to the landing pad of a given hive over a given period of time. A two-tier method for counting bee motions is proposed to estimate levels of omnidirectional bee traffic in videos. The proposed method couples motion detection with image classification. Motion detection acts as a class-agnostic object location method that generates a set of regions with possible objects. Each such region is classified by a class-specific classifier such as a convolutional network, an artificial neural network, or an ensemble of classifiers such as a random forest. The method has been iteratively field tested in BeePi monitors, multi-sensor electronic beehive monitoring systems, installed on Langstroth beehives in real apiaries. Deployment of a BeePi monitor on top of a beehive does not require any structural modification of the beehive’s woodenware, and is not disruptive to natural beehive cycles. In some cases, the proposed method overestimates the human bee motion counts. A detailed analysis of the performance videos shows that a major cause of such overestimation are overlapping motion regions. When several motion points are detected around a single bee, the motion regions cropped around them include the same bee whose motion may be counted several times. Another cause of overestimation are false positives when the second tier misclassifies motion regions containing bee shadows, grass blades, or leaves as bees. In some cases, the proposed method underestimates the human bee motion counts. The underestimation is caused by false negatives, when second-tier classifiers fail to recognize bees or when the motion detection algorithm fails to recognize motion regions of fast-flying bees. Our hand- and auto-designed convolutional networks performed mostly on par with such well-known architectures as ResNet32 and VGG16, which suggests that all training and validation of deep learning models is local. The architectures that do well on large generic datasets are not guaranteed to do well on more specialized datasets. A solid performance of random forests trained on raw images is noteworthy. The accuracies of predicting bee motions with random forests were above 90%. To ensure the replicability of the reported findings and to provide a performance benchmark for interested research communities and citizen scientists, we have made public our curated and labeled image datasets of 167,261 images used to train our second-tier classifiers.

### 2.5. Honey Bee’s Waggle Dance Shows Foraging on Soybeans in Northern Ohio (Poster)

SureshSreelakshmiLinChia-HuaMatchamEmmaRichardsonRodney T.SponslerDouglas B.JohnsonReed M.Department of Entomology, The Ohio State University, Columbus, OH, USA

Honey bees (*Apis mellifera*) depend on floral nectar sources to create the honey they rely on as food. In turn, humans benefit directly from the pollination of insect-dependent crops and other important plants; bees’ work is valued at $20 billion a year. However, there are some plants which may not depend on honey bees for pollination but benefit from their work. One such plant is the soybean, which covered 86.9 million acres of the US in 2018. Soybeans bloom during the late summer, and are one of the few floral resources available to bees at that time. By analyzing the bees’ waggle dance language, we determined that honey bees recruit this nectar source. Bee dances were recorded with a video camera trained on two glass-walled observation hives weekly during soybean bloom, and the videos were processed using FIJI image analysis software. Finally, we plotted locations inferred from bee dances on crop maps to determine where bees are foraging. This information will be used to determine the attractiveness of soybean flowers to bees, and can be further used in order to determine the actual attractiveness of other nectar sources in a given area.

### 2.6. Work in Progress: Using Particle Image Velocimetry to Estimate Incoming, Outgoing, and Lateral Bee Traffic Flows in Videos (Poster)

KulyukinVladimirMukherjeeSarbajitDepartment of Computer Science, Utah State University, Logan, UT, USA

Omnidirectional bee traffic consists of incoming, outgoing, and lateral bee traffic flows. Incoming traffic includes bees flying into the hive, outgoing traffic includes bees flying out of the hive, and lateral traffic consists of bees flying sideways near the landing pad of the hive. The Particle Image Velocimetry algorithm is used to compute incoming, outgoing, and lateral bee traffic flow estimates from videos. These flow estimates are put into temporal sequences over specific time periods (e.g., daily, weekly, bi-weekly, etc.). The temporal sequences are compared with each other with dynamic time warping. Obtained similarity coefficients between temporal sequences are used to estimate how closely incoming and outgoing flows correlate. Consistent misalignments between the flows may indicate abnormal conditions in the monitored colony. Two hundred seventy five 30-s videos from a deployed BeePi monitor were used to evaluate the proposed method. A BeePi monitor is a multi-sensor electronic beehive monitor for Langstroth beehives. Deployment of a BeePi monitor on top of a beehive does not require any structural modification of the beehive, and is not disruptive to natural beehive cycles. The videos were taken 1–14 June 2018. The hive had a new Carniolan bee colony, and was also monitored by a beekeeper who was logging his observations bi-weekly. The hive consisted of a single deep super with a BeePi monitor placed on top of it. On all 14 days on which the videos were taken the monitored hive was healthy in that the queen was alive and laying and the bees were building comb, bringing pollen, and making liquid honey. Each temporal sequence corresponded to a time period from 8:00 a.m. to 8:00 p.m. A total of 14 incoming, 14 outgoing, and 14 lateral temporal sequences were computed. The incoming and outgoing temporal sequences were then compared with dynamic time warping to discover reliable similarity ranges between the incoming and outgoing bee traffic flows in healthy hives.

### 2.7. Honey Bee Foraging in Orchard Landscape in Northern Virginia

SteeleTaylor N.SchürchRogerCouvillonMargaret J.Department of Entomology, Virginia Tech, Blacksburg, VA, USA

Honey bees (*Apis mellifera*) have traditionally been used as pollinators of orchards and food crops, contributing more than $17 billion annually to the US economy. In recent years, the widely-publicized decline of pollinators has been associated with many interconnected factors, including the lack of available forage. This stressor directly and indirectly affects bees, causing both malnutrition and decreases their ability to cope with disease, pests and pesticides. Currently we do not fully understand honey bee food collection, particularly within a fruit crop system, a landscape that is both interesting for its agricultural relevance and its likeliness to represent a feast or famine situation, where forage is predominantly only available when the crop is blooming.

We investigated honey bee foraging in orchards using three observation hives located at the Alson H. Smith Agricultural Research and Extension Center (AREC) in Northern Virginia for the entire foraging season (Spring–Autumn) for two years. We video recorded returning foragers waggle dances then extracted the distance and direction information from the hive to the quality resource in the landscape. We then analyzed these data spatially and temporally to determine where and when bees are and are not foraging in this landscape. Additionally, we will determine both the contribution of orchards to colony forage and, likewise, the contribution of honey bees to fruit pollination. Lastly, a better understanding of foraging in this representative landscape will inform the best management practices for improving food availability to benefit overall pollinator health.

### 2.8. Propolis Exposure Effects on the Honey Bee (Apis mellifera) Mouth Microbiome

DalenbergHollieMaesPatrickMottBrendonAndersonKirk E.SpivakMarlaDepartment of Entomology, University of Minnesota, St. Paul, MN, USA

Honey bees in the wild collect and apply plant resins to the interior of their nest cavity, which is called a propolis envelope. Propolis has been shown to have an antimicrobial activity against honey bee pathogens, but the effect of propolis on the honey bee microbiome is unknown. Honey bees do not consume propolis, but do manipulate propolis with their mouthparts. As honey bee mouthparts are used for collecting and storing nectar and pollen, grooming, and trophallaxis between adults, feeding glandular food to larvae, and cleaning the colony, they are an important interface between the bees’ external and internal environments and serve as a transmission route for core gut bacteria and pathogens alike. We hypothesized that the antimicrobial activity of a propolis envelope would influence the microbial diversity and abundance of the worker mouthpart microbiome. Newly-emerged worker bees were marked, and reared in colonies with or without a propolis envelope. On day nine, the mouthparts were dissected from the marked bees, DNA was extracted, the microbiome was sequenced, and bacterial loads were determined with real-time quantitative PCR. The data revealed that the mouthparts of worker bees in colonies with a propolis envelope had significantly lower bacterial diversity and significantly higher bacterial abundance compared to the mouthparts of bees in colonies without a propolis envelope. Based on the identification of the bacteria, a propolis envelope appears to reduce pathogenic or opportunistic microbes and promotes the proliferation of putatively beneficial microbes, thus restoring the colony microbial ecosystem and positively influencing honey bee health.

### 2.9. Understanding the Sublethal Impacts of Fungicides on Honey Hee Physiology and Nutrition

ChakrabartiPriyadarshiniSagiliRamesh R.Department of Horticulture, Oregon State University, Corvallis, OR, USA

Pesticide-induced lethal and sublethal impacts on bee pollinators are of serious concern in pollination conservation efforts. Pesticide-imposed stress include detrimental impacts on individual bees and the entire colony—ranging from cognitive impairments, flight, and homing disruptions and physiological alterations. Over the last decade, pesticides have emerged as one of the major factors for global bee pollinator declines. What holds added importance is the need to assess the effects of field realistic pesticide exposures. Even though poor nutrition is undoubtedly another important stressor, only a few studies have addressed the underlying, fundamental problems of malnutrition in honey bees, particularly with regard to its interplay with pesticide stress. As honey bee nutrition plays a vital role in mitigating the effects of biotic and abiotic stressors on bees, endeavoring to improve bee nutrition is critical. Pesticides include both insecticides and fungicides and it is important to evaluate their impacts on bee pollinators. There is a gap in knowledge regarding sublethal impacts of sterol biosynthesis inhibitory (SBI) fungicides on bees (especially effects on bee tissue phytosterols) and it is critical to assess such impacts. In the present study, newly-emerged bees were exposed to two SBI fungicides orally. It was observed that exposure to fungicides altered the honey bee tissue phytosterol concentrations and also reduced bee longevity. Further research is needed in the future to explore such impacts.

### 2.10. Feral Bees: Reservoirs of Disease or Traits for Future Breeding?

López-UribeMargarita M.HinshawChauncyEvansKathleen C.RosaCristinaGrozingerChristina M.Department of Entomology, Penn State University, University Park, PA, USA

The feralization of managed honey bees significantly changes selective pressures on colonies. Traits favored in managed colonies, such as high honey production and docility, are not advantageous in wild conditions. On the other hand, unmanaged colonies do not have access to miticide control and supplemental feeding, two of the most important components of beekeeping management to increase the probability of colony winter survival. As a result, most colonies that swarm and establish in wild conditions die within the first year. Previous population genetic studies in the Eastern US indeed suggest that feral colonies are genetically similar to managed colonies suggesting that the origin of many feral colonies are beekeepers’ hives. To better understand host-pathogen dynamics and overwintering success in feral colonies, we have been leading a community science project for three years in Pennsylvania. The goals of the project were to (1) identify the location of feral colonies, (2) estimate over wintering survival, and (3) quantify levels of viral pathogens and immune gene expression comparing feral and managed colonies from the same sites. Our results indicate that feral colonies generally have higher titers of Deformed wing virus (DWV) than managed colonies that indirectly suggests a high *Varroa* mite pressure in feral colonies. Despite the significantly higher pathogen pressure in feral colonies, both groups exhibited similar rates of overwintering success in 2017 and 2018. Through quantitative PCR of pooled samples from 60 colonies, we identified two immune gene biomarkers linked to overwintering survival of feral and managed colonies. Results from this study indicate that feral colonies can be reservoirs of DWV but also have immunological traits linked to viral tolerance that often allows them to overwinter successfully. These immunological traits could be of interest for future breeding programs focused on selecting for genetic lines with adaptive traits for better tolerance to pest and pathogen pressure.

### 2.11. Dancing Honey Bees Communicate Foraging Preferences in Row Crop Production Systems

SillimanMarySchürchRogerTaylorSallyCouvillonMargaretDepartment of Entomology, Virginia Tech, Blacksburg, VA, USA

Agricultural intensification and urbanization contribute to degraded natural lands, increased pesticide use, increased disease spread, and a loss of biodiversity. Bees have been directly and indirectly impacted by these practices because consequences, acute and chronic, are observed on individual and colony-level scales. The compounding nature of these stressors can lead to reduced immunity and, oftentimes, increased mortality. Row crop production systems (e.g., corn, cotton, peanuts, and soybeans) are a subset of agricultural land use. Although these crops are largely wind- or self-pollinated, they do produce pollen and in some cases extrafloral nectaries, which attract flower-visiting insects like bees. Moreover, bees are commonly observed collecting forage in these systems, and honey bees have even been shown to increase cotton yields in cage studies. However, despite the presence of bees in these systems, little is known about their foraging dynamics, such as visitation frequency and seasonality, in this setting. This study will examine how we can use honey bees as bioindicators to assess seasonal nutrition and pesticide exposure of pollinators in row crop systems. To understand more about foraging dynamics, we will decode the honey bee waggle dance, a communication behavior that provides the distance and direction of a resource relative to the hive, to determine when and where honey bees are foraging. We will analyze pollen collected from incoming foragers to assess what they are foraging on throughout the season and additionally test these samples for pesticide residues. Preliminarily, there were significant differences calculated in foraging distance across the season (April to October) (*n* = 8082, χ^2^ = 376.33, *p* < 2.2 × 10^−16^). Longer foraging distances were also observed at the end of major bloom periods. Findings will be used as a basis for providing recommendations to when and where supplemental forage can be most beneficial to bees in this setting and determine how best to deploy pesticides to minimize exposure to pollinators.

### 2.12. Genome-Wide Patterns of Genetic Differentiation of U.S. Commercial Honey Bee Stocks

SaelaoPerotSimone-FinstromMichaelAvalosArianBourgeoisLanieDankaRobertde GuzmanLiliaRinkevichFrankTokarzPhilipUSDA-ARS, Honey Bee Breeding, Genetics and Physiology Lab, Baton Rouge, LA, USA

The genetics of U.S. honey bee stocks remain poorly characterized despite the importance of *Apis mellifera* as a crop pollinator. Several breeding programs have made significant improvements of favorable genetic traits. The variety of bees produced by artificial selection provides an exciting opportunity to explore the landscape of genetic diversity in commonly used stocks. Population genetic analyses found strong genetic similarity among seven stocks, while Pol-line, a stock with mite resistance, showed significant differentiation likely due to strong selective breeding. Juxtaposing the underlying genetic variation of stocks selected for disease-resistance behavior, we identified genes and candidate regions potentially associated with resistance regulated by hygiene. This provides additional evidence for future studies towards understanding the genetic architecture of hygienic behavior. This study provides important insights into the distinct genetic characteristics and population diversity of honey bee stocks used in the United States. Composite signatures of selection helped highlight regions putatively under selection and potentially associated with disease resistance behavior. This study presents the initial effort towards effectively cataloging the standing variation within widely-used honey bee stocks.

### 2.13. Measuring Honey Bee Utilization of Conservation Reserve Program (CRP) Pollinator Planting Using DNA Metabarcoding

McMinn-SauderHarper^1^RichardsonRodney^1^SmithMike^2^JohnsonReed^1^1Department of Entomology, The Ohio State University, Columbus, OH, USA2Conservation Technology Information Center, West Lafayette, IN, USA

Since its introduction in the 1980s to help improve soil quality on agricultural land, the Conservation Reserve Program (CRP) has had a number of positive ecological effects, including reduced habitat fragmentation and increased natural forage for pollinators. One present goal of CRP management is to increase pollinator presence and diversity by planting native seed mixes that promote foraging. The pollinator seed mix is composed of species that flower throughout the season to ensure that foraging pollinators have sufficient pollen and nectar resources at each point of the year. This high quality habitat provides an excellent opportunity to study honey bee nutrition and identify if CRP planting influences honey bee pollen collection. This study aims to highlight the primary sources of honey bee forage on CRP land in the northern Midwest as well as to assess honey bee utilization of the floral resources provided by the pollinator seed mix. Using citizen science methods, we received pollen trapped samples from beekeepers in Ohio, South Dakota, Indiana, Illinois, and Michigan. Pollen homogenization and metabarcoding methods were used to analyze and quantify pollen collected at different points in the season. Results indicate that honey bees located near CRP frequently use major mass flowering resources such as *Glycine*, *Trifolium*, and *Symphiotrichum* for foraging throughout the season. In addition, results indicate moderate use of flowers in the pollinator seed mix by bees located near CRP. These results have implications for land and colony management as well as pollinator seed mix design.

### 2.14. Spray Adjuvant Interactions with Insecticides and Fungicides Applied to California Almonds

JohnsonReedWalkerEmilyLinChia-HuaDepartment of Entomology, The Ohio State University, Columbus, OH, USA

Beekeepers providing honey bees for almond pollination continue to experience unacceptable losses that they attribute to the exposure to insecticides, fungicides, and spray adjuvants applied during almond bloom. Some beekeepers observe the death of adult bees, larvae, or pupae while others report queen problems or experience lingering issues in colonies weeks after almond pollination. Data from the California Department of Pesticide Regulation shows that in 2017 insecticides were applied to approximately 55,000 acres of almonds during the blooming period (15 February–15 March) when bees were present. Nearly all insecticides applied during this period were in a tank-mix with fungicides and spray adjuvants. Previous work identified the combination of the insecticide Altacor^®^ (chlorantraniliprole) and the fungicide Tilt^®^ (propiconazole) as potentially toxic to adult and larval honey bee workers. We found that the addition of the spray adjuvant Dyne-Amic^®^ to this combination, applied in a spray to bees at the maximum label rate, resulted in substantial mortality of adult bees. Worker larvae that were artificially reared with diet contaminated with the active ingredients in these pesticides also showed reduced survival. Diflubenzuron, the active ingredient in Dimilin^®^, has demonstrated toxicity to honey bee larvae alone and in combination with fungicides. Further increased toxicity was observed when the spray adjuvant was added to diflubenzuron in larval diet. Spray adjuvants have the potential to further increase the toxicity of insecticides and combinations that are already toxic to honey bees.

### 2.15. A Widely-Used Fungicide Produces Symptoms of Colony Collapse Disorder in Honey Bees (Apis mellifera)

FisherAdrianIIUSDA-ARS, Tempe, AZ, USA

Honey bee (*Apis mellifera*) and other pollinator populations are declining worldwide for unexplained reasons, threatening over $12 billion in agriculture that depends on pollination services. Fungicides are applied to prevent rot diseases while many crop plants are in bloom, leading to wide consumption by pollinators. Field colonies of honey bees were forced to feed on pollen containing Pristine^®^, composed of the fungicides boscalid and pyraclostrobin, at four doses ranging from 0.1 to 100× levels previously reported for agricultural pollen. Pristine^®^ consumption produced the symptoms of colony collapse disorder, reducing colony adult populations in a dose-dependent manner with foragers dying outside the hive, and reducing over-winter survival. Pristine^®^ consumption lowers colony populations by causing workers to forage and die earlier. Pristine^®^ consumption reduced forager associative learning abilities, potentially reducing pollination efficiency and contributing to “lost foragers”. Pristine^®^ increased colony pollen foraging and storage, suggesting it may act by interfering with protein digestion or absorption, perhaps by inhibiting intestinal mitochondria. Together, these findings suggest that fungicides play a significant role in pollinator decline and that the safety of fungicides for pollinators must be re-evaluated. This research was supported by USDA 2017-68004-26322.

### 2.16. Assessing Non-Woven Cleaning Pads as a Control Technique for Small Hive Beetle (*Aethina tumida*) Infestations in Honey Bee Colonies (Poster)

PolleeAdriannaSureshSreelakshmiJohnsonReedDepartment of Entomology, The Ohio State University, Columbus, OH, USA

Small hive beetles (SHB), *Aethina tumida*, are a common pest found within many European honey bee (*Apis mellifera*) hives in the US. The integrity of harvestable honey and the health of honey bee colonies are often at risk when large SHB infestations build up, which can result in economic losses for beekeepers. This study examined the potential for unscented non-woven cleaning pads, balled up and placed under the inner cover, to be used as both a monitoring and a control method for SHB. A standard method for monitoring SHB populations in colonies is a corrugated plastic strip placed on the bottom board. We placed both plastic strips and non-woven pads inside hives to evaluate the relative effectiveness of each monitoring approach. Additionally, non-woven pads were left in colonies for six weeks to evaluate SHB populations in the presence or absence of the pads. Overall, the corrugated strips failed to capture as many SHB as the non-woven pads, and the total number of beetles trapped in pads showed a general decrease in apiaries where pads were maintained. The use of non-woven pads for management of SHB offers beekeepers a new minimally invasive, non-chemical, and cost-effective method of control for SHB.

### 2.17. Deformed Wing Virus Variant Populations Shift During Transmission Mimicking Varroa Mites

RayAllyson M.DavisSheldon L.RasgonJason L.GrozingerChristina M.Department of Entomology, Pennsylvania State University, University Park, PA, USA

Parasites and pathogens of honey bees are two primary contributors to declining bee health. Uncontrolled infestations of parasitic *Varroa destructor* mites, and the main virus it vectors, deformed wing virus (DWV), drive winter losses in temperate regions. Previous research suggests that transmission of DWV through *Varroa* mites results in increased viral loads, changes in the frequency of certain viral strains, and increased host mortality. Understanding the mechanism underlying this *Varroa*-mediated shift in DWV dynamics is critical for mitigating negative impacts on this critical pollinator species, as well as providing a model for understanding how vectors influence pathogen evolution. To evaluate how *Varroa* transmission influences DWV dynamics and evolution, we serial passaged a population of DWV by injection through in vitro reared honey bee pupae and measured viral populations throughout the five passages. Our results suggest that repeated pupal-to-pupal transmission, mimicking iterative vectoring by *Varroa*, does alter the proportions of variants in viral populations, possibly simulating the selective pressure imposed by a viral vector and serving as a mechanism for virus evolution.

### 2.18. Horizontal Transmission of Deformed Wing Virus (DWV) in Honey Bee Colonies

KevillJ.L.LeeK.GoblirschM.McDermottE.TarpyD.R.SpivakM.SchroederD.C.Department of Veterinary Population Medicine, University of Minnesota, St. Paul, MN, USA

Pathogen transmission from workers to queens is rarely examined. During the queen’s life, she will be tended by attendant worker bees who will feed and groom her. Viruses have been detected in salivary secretions, honey, beebread, pollen and royal jelly. Therefore, the queen may be exposed to pathogens by the activities of her attendants, yet she is long-lived compared to workers. To address this question, we looked at pathogen transmission from workers to queens. The queens of 42 colonies were exchanged into honey bee colonies, upon initial exchange worker bees were analyzed for pathogens and then the queens were culled 24 days after the exchanged. Samples were screened for Deformed wing virus master variants (DWV-A, DWV-B, and DWV-C), Black queen cell virus (BQCV), Acute bee paralysis virus (ABPV), Chronic bee paralysis virus (CBPV), Israeli acute paralysis virus (IAPV) Lake Sinai virus (LSV), Nosema, and Trypanosomes. We found that colonies were negative of BQCV, ABPV, CBPV AND DWV-C. The most abundant pathogens in the study were LSV, Nosema and DWV-B with 50%, 44%, and 40% prevalence, respectively. All workers were LSV positive and 89% Nosema positive, whist all queens were negative. DWV variants were detected in both workers and queens, yet transmission of DWV did not occur from worker to queen.

### 2.19. How Many Mates Are Enough? Toward Understanding the Relationship between Queen Mating Number and Colony Fitness

HaganKatherine L.DelaplaneKeith S.University of Georgia, Athens, GA, USA

The evolution of polyandry is an important event in the natural history of the western honey bee, *Apis mellifera* L., and many hypotheses have been put forth to explain its constraints and adaptive value at the colony level. One such set of hypotheses are the genetic variance hypotheses, which suggests that higher levels of polyandry provide genetic diversity to the colony, contributing to colony survival and success. Indeed, many field studies have indicated that there is a positive correlation between queen mating frequency and colony performance. However, these studies have compared queens within narrow ranges of mating categories, and our knowledge none has attempted to study a wider range of polyandrous conditions. In this study, we sought to characterize the regression relationship between mating number and colony fitness across a wide range of polyandrous conditions. Utilizing artificial insemination, we mated honey bee queens the semen of 1, 2, 4, 8, 16, and 32 males to create six levels of polyandry. Subsequently, we tracked their colonies for various measures of fitness, including honey production, pollen collection, brood production, and mite number. Analysis of our incomplete (at press time) data indicate significant differences in the number of mites and brood production across our polyandry treatments. These preliminary results bolster previous confirmations of the benefits of polyandry and inform our attempts at understanding the relationship between the mechanisms underlying queen mating number and colony fitness.

### 2.20. Testing a Queen Vaccine against Chalkbrood Infection (Poster)

GoblirschMichaeal^1^FreitakDalial^2^1Department of Entomology, University of Minnesota, St. Paul, MN, USA2Institute of Biology, University of Graz, Graz, Austria

Insects retain information about past exposure events to pathogens through interactions with the cell walls of bacteria and fungi (e.g., pathogen-associated molecular patterns, peptidoglycans, and lipopolysaccharides). These immune elicitors are used by the insect adaptive immune system to develop long-term resistance against subsequent exposure. Insects can also transfer immune elicitors to offspring through a process called transgenerational immune priming (TgIP), resulting in increased resistance and survival of offspring. Transgenerational immune priming has been documented in honey bees. For example, larvae from queens exposed to heat-killed spores of *Paenibacillus larvae* (American foulbrood) have greater priming of immune cells and reduced mortality after subsequent challenge with the pathogen. Understanding the mechanism by which honey bee queens deliver information about pathogens to their offspring has led to the development of an edible vaccine that can be given to queens. We conducted a field trial where we exposed queens to an oral vaccine against *Ascosphaera apis* (chalkbrood) or sham vaccine and reintroduced them into colonies. We then challenged offspring of queens with sucrose solution containing 1% ground chalkbrood mummies or sucrose solution alone. We present preliminary findings of the effects of vaccination on the egg-laying ability of queens and whether there was an increase in fitness-related traits (i.e., emergence success and adult bee longevity) for offspring of vaccinated queens following exposure to *A. apis*.

### 2.21. The Heat Shock Response Is Antiviral in Honey Bees (Apis mellifera)

McMenaminAlexander J.DaughenbaughKatie F.FlennikenMichelle L.Department of Microbiology and Immunology, Montana State University, Bozeman, MT, USA

Since 2006, honey bee populations in some parts of Europe and North America have experienced high annual losses that are associated with elevated pathogen prevalence and abundance. The majority of honey bee pathogens are positive-sense single-stranded RNA viruses, therefore, a better understanding of honey bee-RNA virus interactions at the individual and cellular levels may result in the development of strategies that mitigate colony losses. In previous transcriptome-level studies, we determined that the expression of heat shock proteins (HSPs) in virus-infected bees at 72 h post infection was greater comparted to mock-infected bees. HSPs and the heat shock response (HSR) help maintain protein homeostasis (proteostasis), especially in response to various stressors like extreme changes in body temperature and viral infection. Heat shock proteins have been shown in *Drosophila* to play an important role in the function of RNA interference machinery, which mediate a nucleic acid-based antiviral response. Therefore, we hypothesized that the HSR is important for combatting viral infection in worker honey bees. To test that hypothesis, individual age-matched bees were micro-injected with a model virus Sindbis-GFP (SINV-GFP), recovered, and then subjected to heat shock (i.e., 42 °C for 4 h). Quantitative-PCR (qPCR) analysis of SINV-GFP genome equivalents revealed a significant decrease in viral abundance in heat-shocked bees. Although, virus abundance was lower in heat-shocked bees the expression of two HSPs (Hsc-70-3 and Hsp83-like) was not increased beyond the greater levels of expression in all virus-infected bees compared to mock-infected bees. Unexpectedly, heat-shock alone induced expression of an immune gene, MF116383, but not expression of argonaute-2 or dicer-like. Ongoing studies will further elucidate the mechanism(s) of MF116383 and HSR-mediated responses in limiting virus infection in honey bees.

### 2.22. Dancing Honey Bees Communicate Forage Availability in Representative Landscapes in Virginia

OhlingerBradley D.SchürchRogerCouvillonMargaret J.Department of Entomology, Virginia Tech, Blacksburg, VA 24061, USA

Pollinator declines have inspired public activism and have fueled efforts to better manage their populations. Unfortunately, we lack evidence-based solutions to pollinator issues that are effective and easy to implement. In response, private, commercial, and government efforts have begun to provide supplemental foraging habitat for pollinators by planting additional flowers. However, such efforts must consider the temporal and spatial dynamics of foraging habitat and the phenology of pollinator attractive flower species against the nutritional needs of the pollinators. In this study, we monitored the waggle dances of honey bees foraging in a mixed-use landscape in Blacksburg, VA to (1) determine the types of foraging habitat that honey bees prefer to visit and to (2) identify monthly fluctuations in the availability of honey bee foraging habitat. To address these objectives, we decoded waggle dances to map the association between National Land Cover Database land cover categories and honey bee foraging dynamics. Additionally, we used the distances communicated by waggle dances as a proxy for forage availability, with increases in foraging distances representing probable decreases in the availability of honey bee forage. Our analysis shows high visitation rates in areas with a high proportion of hay meadows and low visitaton rates in areas with a high proportion of forest. Additionally, our results showed relatively stable monthly median foraging distances, ranging from 0.68 km in August to 1.61 km in June. However, even within this diverse landscape, there were temporal fluctuations in communicated foraging distances, as a proxy of availability, with foraging honey bees communicating a 53% and 77% increase in median foraging distance in June and October, respectively. When compared to previous studies conducted in other landscape types, our results suggest that this mixed-use landscape provided sustained foraging habitat throughout the honey bee foraging season with a possible small decrease in forage availability in June and October. These months likely pose a challenge for local pollinators and should be targeted by planting flower species with appropriate phenology. Taken together, these results provide insight into honey bee foraging dynamics in a mixed-use landscape and demonstrate the potential use of waggle dances as bioindicators to assess pollinator habitat quality and to guide land management efforts.

### 2.23. Stable Carbon and Nitrogen Isotope Ratios Elucidate Nutrient Dynamics in *Nosema*-Infected Worker Bees

WebsterThomas^1^KammingaKatherine^1^MunizziJordon^2^1College of Agriculture, Kentucky State University, Frankfort, KY, USA2Department of Earth and Environmental Science, University of Kentucky, Lexington, KY, USA

*Nosema ceranae* infection in honey bee workers was studied by the measurement of stable carbon and nitrogen isotopes. This intracellular parasite rapidly dominates the bee’s midgut epithelium, assuming most of the volume of infected cells (see Figure 2). Worker bees were caged and maintained at 32 °C and fed either 50% sucrose in water with *Nosema* spores, or 50% sucrose without spores. After 10 days, worker bees were removed from the cages, and midguts were dissected from infected and uninfected bees. Spores were separated from midgut host tissue by centrifugation. Samples of spore pellets and host tissue supernatant were each homogenized and dried. To determine the ratios of 12C to 13C, and of 14N to 15N, samples were analyzed using a Thermo EA IsoLink elemental analyzer (EA) and Thermo MAT 253 isotope ratio mass spectrometer. Raw δ15N and δ13C values were normalized to the international VPDB and AIR-N2 scales, respectively, using USGS40 and USGS41.

We found significant partitioning of the nitrogen isotopes, with higher δ15N for the supernatant (the midgut host tissue) than the spores (*p* < 0.01). In other words, relatively less 15N was detected in the spore pellet compared to the midgut host tissue. This indicates aggressive incorporation of nitrogen into the rapidly developing *N. ceranae*. For the carbon isotopes the reverse effect was found. The δ13C values for the supernatant were less than those for the spores (*p* < 0.01). These values show that the developing *N. ceranae* do not aggressively incorporate the carbon. In fact, the heavier isotope of carbon is preferentially acquired by the spores, apparently because the midgut acquires more carbon for growth and respiration. These results pertain to recent studies (e.g., Porrini et al., 2011 Apic. Res. 50: 35–41) indicating that pollen consumption by worker bees exacerbates *N. ceranae* infection.

### 2.24. Influence of Pollinator Plantings on Honey Bee Health

LeeKatieHerron-SweetChristina R.FriedrichKileySpivakMarlaCariveauDanielDepartment of Entomology, University of Minnesota, St. Paul, MN, USA

Floral resources are critical to sustaining healthy populations of pollinators and other beneficial insects. Declines in managed bee health and wild pollinator populations have prompted numerous government and non-government groups to promote and implement pollinator habitat enhancements throughout the United States. However, critical research gaps exist in how to provide effective, economically-feasible floral resources for multiple groups of pollinators and other beneficial insects. We established a landscape-scale experiment to rigorously quantify how local and landscape factors influence the success of pollinator plantings for honey bees, native bees, and natural enemies. To examine the effects of plantings on honey bee health, we manipulated the landscape context (percent natural area) and plot size of experimental plantings at 27 sites across ten counties in Southwestern Minnesota. Locations were chosen to fit a low (1–9%), medium (10–29%), or high (>30%) amount of natural area. Plots at the locations were small (1–4 acres), large (8–16 acres), or control sites with no planting. Each plot size category had three replications within each percent natural area category. We maintained seven honey bee colonies at each location to determine the influence of plot size and percent natural area on the amount of incoming pollen, pollen lipid and protein content, colony weight, adult bee and brood populations, honey production, pest and disease levels, vitellogenin levels, and colony mortality. The results can enable land managers to better prioritize placement and planning of floral plantings to generate a higher return on investment, especially given increasing costs of creating pollinator habitat.

### 2.25. Investigating Strategies to Limit Virus Infection in Honey Bees

ParekhFenaliFaurot-DanielsCayleyDaughenbaughKatie F.SheppardWalter S.ChakrabartiPriyadarshiniSagiliRameshFlennikenMichelleMontana State University, Bozeman, MT, USA

Since 2010, US honey bee colony losses have averaged 38% annually. Colony losses are associated with several abiotic and biotic factors including virus infection. Currently, there are no antiviral treatments for honey bee viruses. Recent studies suggest that feeding bees fungal extracts, thyme oil, and protein-containing diets reduce the impact of viral infections in honey bees possibly by priming immune responses and/or promoting overall bee health. To further investigate the role of supplements and diet on the outcome of virus infection in honey bees, we performed laboratory-based honey bee virus infection assays using a panel of viruses (i.e., Sindbis-GFP, Flock house virus [FHV], and Deformed wing virus [DWV]) and monitored honey bee survival and virus abundances up to four days post-infection. Virus-infected bees were fed with extracts from mycelium of three fungal species (i.e., *Fomes fomentarius*, *Ganoderma lucidum*, or *Laricifomes officionalis*), or thymol, a phytochemical derived from thyme, or protein diets (i.e., multifloral pollen mix, UltraBee^®^ Mann Lake, artificial diet formulated by Oregon State University (OSU), OSU diet supplemented with 0.5% dry diet weight of 24-methylenecholesterol, a vital phytosterol and micronutrient). Our data indicate reduced mortality for bees infected with SINV-GFP or DWV and fed either *Fomes fomentarius* extract or thymol. There was a significant reduction in viral load in thymol fed bees infected with SINV-GFP. FHV-infected bees fed either fungal extracts did not show reduction in viral load. The impact of protein supplementation on virus infection varied by virus. There was a significant reduction in viral load in SINV-GFP-infected bees fed UltraBee^®^ or OSU formulated diet compared to levels of SINV-GFP in bees fed only 50% sucrose. FHV-infected bees fed either diet did not show reduction in viral load. For some experimental groups we also assessed the expression of key honey bee antiviral genes, including dicer, ago-2, and MF116383 and determined that the expression of dicer and ago-2 was greater in virus-infected bees compared to the mock-infected bees. Though the data in these studies were variable and less promising than previous results, in the long-term this line of investigation is important for the development of strategies that beekeepers may adopt to reduce virus-associated colony losses.

### 2.26. Effects of Imidacloprid-Contaminated Honey Stores on Queen and Worker Activities in Fall and Winter Colonies

CarrollMark J.BrownNicholasUSDA-ARS, Carl Hayden Bee Research Center, Tucson, AZ, USA

Neonicotinoid-contaminated sugar stores can have both near term and long term effects on honey bees due to their persistence in honey stores. Lower Sonoran desert colonies were given treatment sugar syrups containing 0 ppb, 20 ppb (field relevant), or 100 ppb (above field relevant) imidacloprid over six weeks to simulate a contaminated early fall nectar flow. Colonies were evaluated immediately after (post-treatment) and 10 weeks after (mid-winter) the treatment phase to compare immediate and latent effects of neonicotinoid-contaminated sugar. During post-treatment evaluations, 0 ppb colonies had larger adult populations and more brood than 20 ppb or 100 ppb colonies, while mid-winter 0 ppb and 20 ppb colonies had larger adult populations than 100 ppb colonies. Colonies experienced seasonal declines in stored pollen accumulation but no treatment effects. Lower colony performance among 100 ppb colonies was associated with reduced worker effort rather than adult lifespan. RFID tracking of individual workers revealed that workers from different treatment groups had similar adult lifespans; however, 100 ppb workers engaged in external hive activities (including foraging) less often than 0 ppb workers. High levels of imidacloprid exposure affected queen but not worker nutritional physiology. Nest workers appeared to retain well-developed hypopharyngeal glands (as indicated by soluble head protein) across treatments and time points. Mid-winter queens from 0 ppb colonies had marginally higher ovary protein than queens from 100 ppb colonies and more ovary lipids than queens from 20 ppb colonies. Notably, however, queen nutrient stores in non-reproductive tissues (fat bodies) did not differ across treatment groups. Queens from different treatment groups were attended by comparable numbers of retinue workers and had similar mandibular gland contents of four QMP (queen mandibular pheromone) components essential to queen retention and queen care. These results indicate that very high levels of imidacloprid exposure in nectar stores can negatively affect colony performance well after initial collection and storage.

### 2.27. Identifying Food-Safe Antimicrobial Compounds to Control European Foulbrood (Melissococcus plutonius) in Honey Bees (Poster)

KurkulColinMurrayStephanieJohnsonReedDepartment of Entomology, The Ohio State University, Columbus, OH, USA

European honey bees (*Apis mellifera*) face a variety of fungal, bacterial, and viral pathogens which are detrimental to colony survival. One of these bacteria, *Melissococcus plutonius*, causes a disease called European foulbrood which affects larvae and can cause infected larvae to die from starvation. For many years oxytetracycline, an antibiotic, was used to control *M. plutonius*, but the availability and usefulness of this drug has been eroded by regulatory changes, concerns about honey contamination and the possibility of selection for antibiotic resistance. Honey bees, however, produce their own antimicrobial substances, including propolis, which is a mixture of saliva, beeswax, and plant resins. Propolis has demonstrated antimicrobial properties, likely derived from plant resin constituents, and is thought to play a role in promoting colony health. In addition to propolis extracts, this study screened a suite of plant-derived compounds to determine their potential to control *M. plutonius*. Testing was performed using in vitro rearing of honey bee larvae, as well as minimal inhibitory concentration (MIC) microbial assays to test the effectiveness of these compounds as antimicrobial agents. Our research suggests plant resin constituents may be effective against *M. plutonius* and can be tolerated by larval honey bees.

### 2.28. LncRNA Profile in Honey Bee (*Apis mellifera*) Female Larvae and Its Function on Caste Determination

YinLing^1^,^2^LuGuangfu^1^ZhangHuanxin^1^ChenYuyong^1^HuangZachary Y.^2^WangKang^3^JiTing^3^ChenGuohong^3^1Jiangsu Agri-Animal Husbandry Vocational College, 8 East Phoenix Road, Taizhou 225300, Jiangsu, China2Michigan State University, 288 Farm Lane, East Lansing, MI 48824, USA3Yangzhou University, 88 South University Ave., Yangzhou 225009, Jiangsu, China

In honey bees (*Apis mellifera*), the queen and workers are genetically identical, however they show striking differences in morphology, behavior, and lifespan. Female larvae are “totipotent” in the sense that they can either develop into queens or workers depending on the environment. If worker bee larvae younger than three days are transplanted to queen cells, the worker larvae will develop into queens with mature ovaries, and the earlier the larvae are transplanted the better the queen quality (e.g., more ovarioles). Long noncoding RNAs (lncRNAs) have emerged as significant players in almost every level of gene function and regulation. In this study, we tried to find the role of lncRNAs in the development of the larvae under different transplanted ages. Worker larvae from a single-cohort colony were transplanted into queen cells at 1st day (L1), 2nd days (L2), and 3rd days of age (L3), and then RNA-Seq method were used to monitor the expression files of the lncRNAs of four-day-old larvae. Nine libraries were generated by three biological reolicates. A total of 2063 novel lncRNAs, 152 differentially expressed lncRNAs between L1 and L2, 255 between L1 and L3, and 95 between these two comparisons. Bioinformatic analysis showed that lncRNAs differentially expressed between L1 and L2 were mostly involved in amino acids metabolism, meanwhile, lncRNAs differentially expressed between L1 vs. L3 were mostly involved in longevity regulating pathway and protein processing in endoplasmic reticulum. Our results indicated that the later the larvae are transplanted the more lncRNAs are differentially expressed, and the lower the queen quality, especially for life span which is perhaps the largest difference between queen and workers.

### 2.29. Factors Affecting European Foulbrood Risk and Recovery

MilbrathMeghan O.AndersonKirkEvansJayGrahamKelseyKevillJessicaMottBrendonQunlanGabrielaSchroederDeclanIsaacsRufusDepartment of Entomology, Michigan State University, Lansing, MI, USA

For decades, beekeepers have been concerned about high rates of European foulbrood (EFB) disease in honey bee colonies used in blueberry pollination. Historically, EFB was thought to be relatively benign, with spontaneous resolution in most hives. In recent years, however, many beekeepers report that the disease may be staying longer in the colonies, presents with different signs, and that it is more severe, often leading to colony death. In 2018 and 2019 we followed over 100 colonies in blueberry pollination to determine the incidence of EFB disease and determine risk factors for disease development and progression. We followed colonies that were in three types of blueberry fields (conventional, organic, and no-spray/unmanaged). We evaluated the benefit of providing protein supplementation during bloom, and evaluated the quantity and diversity of incoming pollen. We found colonies were bringing in a large amount of diverse pollen, and did not see a consistent benefit to additional pollen supplementation. We tested the pollen during blueberry bloom for multiple pesticides, and found that bees were exposed to a wide variety of agrochemicals at this time. We found that about 30% of the colonies developed EFB disease in 2018, and almost 60% developed EFB in 2019. In both years, we followed subset of colonies to identify the rate of recovery, and for atypical presentation, and found many colonies with prolonged infections, some lasting well into August. EFB was confirmed microscopically in every hive, and six additional hives with varying presentation each year were further sampled to identify the microbiome of the nurse bees, and 58 hives were sampled for virus presence in 2019. We found high levels of both DWV-A and -B in colonies with signs of EFB, even in the absence of *Varroa* mites.

### 2.30. Targeting Potassium Ion Channels to Enhance Honey Bee Immune Health

SwaleDaniel R.FellowsChristopher J.AndersonTroy D.Department of Entomology, Louisiana State University, Baton Rouge, LA, USA

The beekeeping industry has experienced unsustainable losses of managed colonies, which has been partially attributed to reduced immunocompetence that leads to acute virus outbreaks and bee mortality. To address these colony losses, we have focused on the identification of novel physiological pathways that can increase immune function and decrease virus-mediated mortality at the individual and colony level. Prior studies have shown that the pharmacological activation of ATP-sensitive inward rectifier potassium (KATP) channels improves survivorship in honey bees during infection with a model virus, indicating these channels may play a role in the regulation of antiviral immunity. In addition, KATP channels are known to be coupled to the metabolic state of the cell and are likely regulators of reactive oxygen species (ROS), which play crucial roles in the immune regulation during viral infection. Therefore, the aim of this study is to validate the functional relationship between KATP channels, ROS, and survivorship during viral infection honey bees. Our data indicate that during paraquat-induced oxidative stress, pretreatment with pinacidil, a known KATP channel activator, is capable of significantly reducing total antioxidant levels, indicating that KATP channels can regulate ROS production. Further, we have shown that treatment with pinacidil increases survival of bees while decreasing viral replication following infection with Israeli Acute Paralysis Virus, which is a virus known to cause significant honey bee mortality in beekeeping industries. Taken together, KATP channels are functional regulators of ROS generation in honey bees and provide a significant link between ROS and antiviral immune response in honey bees. These data address fundamental questions regarding honey bee immune responses and provide putative mechanisms to enhance pollinator health.

## Figures and Tables

**Figure 1:**
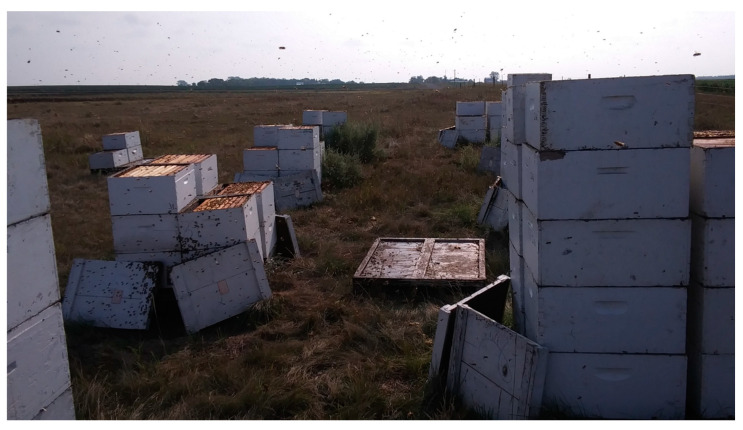
Honey extraction from sample colonies during the study, South Dakota. Yields were quantified to determine differences based on stock, location, and year.

**Figure 2:**
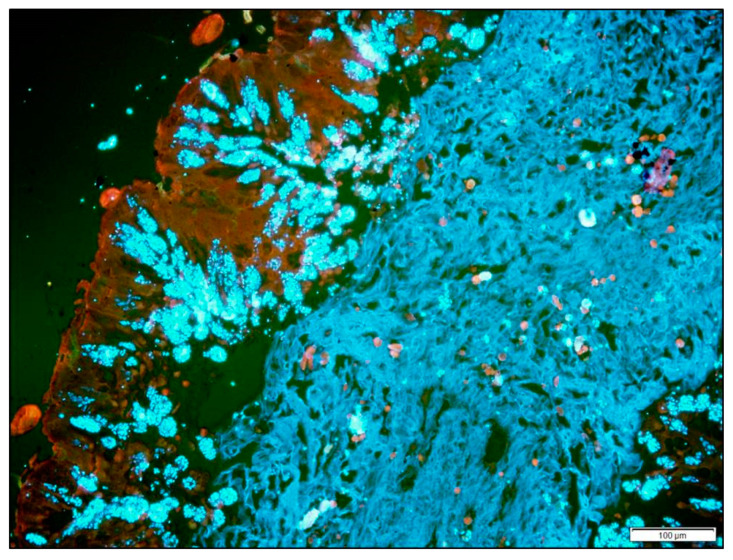
The worker bee midgut becomes heavily infected with *Nosema ceranae* 10 days after spore ingestion. Mature spores fluoresce blue-white on staining with calcofluor. Midgut host cells are red, and the peritrophic matrix within the midgut lumen is light blue. Micrograph by Katherine Kamminga.

